# Aortic Relaxant Activity of *Crataegus gracilior* Phipps and Identification of Some of Its Chemical Constituents

**DOI:** 10.3390/molecules191220962

**Published:** 2014-12-15

**Authors:** Abigail Hernández-Pérez, Moustapha Bah, César Ibarra-Alvarado, José Fausto Rivero-Cruz, Alejandra Rojas-Molina, Juana Isela Rojas-Molina, José Alejandro Cabrera-Luna

**Affiliations:** 1 Posgrado en Ciencias Químico Biológicas, Facultad de Química, Universidad Autónoma de Querétaro, Centro Universitario, Cerro de las Campanas, Querétaro 76010, Qro., Mexico; 2 Departamento de Farmacia, Facultad de Química, Universidad Nacional Autónoma de México, Circuito exterior, Coyoacán 04510, Mexico D.F., Mexico; 3 Facultad de Ciencias Naturales, Universidad Autónoma de Querétaro, Campus Juriquilla, Avenida de Las Ciencias S/N, Delegación Santa Rosa Jáuregui, Querétaro 76230, Qro., Mexico

**Keywords:** *Crataegus gracilior*, aorta, vasorelaxation, phenolics, triterpenes

## Abstract

This study focused on the assessment of the vasorelaxant activity of the organic and aqueous extracts obtained from leaves and fruits of a Mexican hawthorn (*Crataegus gracilior*) on isolated rat aorta, and on the purification and identification of some of their secondary metabolites by the use of chromatographic and spectroscopic techniques. The results obtained showed that the methanol extract has a significantly more potent and effective vasorelaxant effect than the other tested extracts, with an EC_50_ = 8.69 ± 4.34 µg/mL and an E_max_ = 94.6% ± 11.30%, values that are close to that of acetylcholine, the positive control. From the same extract, two major triterpenes were isolated and identified as ursolic and corosolic acids by comparison of their experimental NMR spectroscopic data with those reported in the literature. Chlorogenic acid, rutin, quercetin, kaempferol and (+)-catechin were also identified using HPLC coupled with PDAD. All these compounds have already been proven to possess on their own antihypertensive effect and other benefits on cardiovascular diseases and they can support, at least in part, the traditional use of this plant species.

## 1. Introduction

In Mexico, as in many other countries, cardiovascular diseases are the main cause of death among adults [1]. Hypertension (HT), the so-called “silent killer”, is an important factor that triggers such diseases. More than a third of the Mexican population is now suffering from HT [2]. This is mainly due to the worldwide invasive and inadequate lifestyle adopted in the last decades. Despite some efforts to control the propagation of cardiovascular diseases, these continue to rise. The current standard drug therapy for HT worldwide makes use of six classes of chemical agents that mainly differ from one another by their mechanisms of action [3]. However, these conventional medicines are both expensive to most of the patients suffering from HT and have undesired side effects. Alternative resources, and most likely the most used around the world to prevent and/or treat these diseases, are medicinal and edible plants. Although there have been reports of many intoxications from the regular consumption of certain medicinal plants, they continue to be not only the source of biologically active principles, but also the most available resource to people to treat or prevent many diseases, including those considered chronic-cancer, cardiovascular, diabetes. Both facts support the need to assess their chemical contents, pharmacological activity and their safety. For cardiovascular diseases, plants of the genus *Crataegus* are among the most widely used. All Mexican *Crataegus* species are employed for roughly the same medicinal purposes.

*Crataegus* is a genus widely distributed around the world and many of its species are employed in traditional medicine to treat cardiovascular diseases, mainly due to their contents of phenolic compounds. Fruits of any of the species grown in Mexico are eaten fresh and their extracts obtained by decoction are also consumed both as hot beverages and for medicinal purposes to treat cough, asthma, bronchitis, and as diuretic agents. The leaves, fruits and flowers are frequently employed to modulate contractions induced by tachycardia and to improve coronary blood flow [4]. To be useful to the consumers, these plants must contain suitable chemical constituents and possess ascertainable pharmacological properties. In fact, there are many standardized extracts obtained from leaves and flowers of *Crataegus* spp*.* that are nowadays marketed in Europe and Asia for the treatment of some cardiovascular diseases. The best known—WS1442^®^ and LI132—have being standardized on the basis of either their oligomeric procyanidins [5,6] or simple flavonoids which are estimated as equivalents of hyperoside [7] or vitexin [6]. These preparations are used both as dietary supplements and for therapeutic purposes where they are usually employed as adjuncts to standard therapy. Clinical studies have proven their safety when taken at recommended doses [8,9]. Besides the phenolics, many terpenoids have also been identified in *Crataegus* species [10]. Fifteen out of the fully identified *Crataegus* species around the world are grown in Mexico [11]. They are all commonly known as “tejocote”. This local name is derived from the Náhuatl word “te-xocotl” that means “tough fruit”. Ten of these species are endemic in Mexico [11]. *C. gracilior* Phipps (formerly known for a long time as *C. pubescens*) is found in the center of Mexico, including the state of Querétaro which is one of the richest distribution zones [12]. Of the Mexican *Crataegus* species, there is only one study that reports the vasorelaxant effect of extracts of *C. mexicana* on guinea-pig trachea [13], despite the fact that most of these plants are also traditionally used for cardiovascular disturbances like those implicated in coronary circulation, tachycardia and hypertension [4]. Chemical studies of the flowers of three of the Mexican species have reported four quercetin glycosides [14]. The mechanisms by which some flavonoids and triterpenes already found in *Crataegus* exert their effects on cardiovascular system have been elucidated [15,16,17].

The present study aimed to investigate the *ex vivo* rat aortic vasorelaxant activity of the organic extracts of the leaves and the aqueous extracts of the fruits of *Crategus gracilior*, in order to determine if this medicinal plant truly possesses the desired vasorelaxant effect, and to identify some of its bioactive chemical compounds.

## 2. Results and Discussion

### 2.1. Pharmacological Evaluation

**Figure 1:**
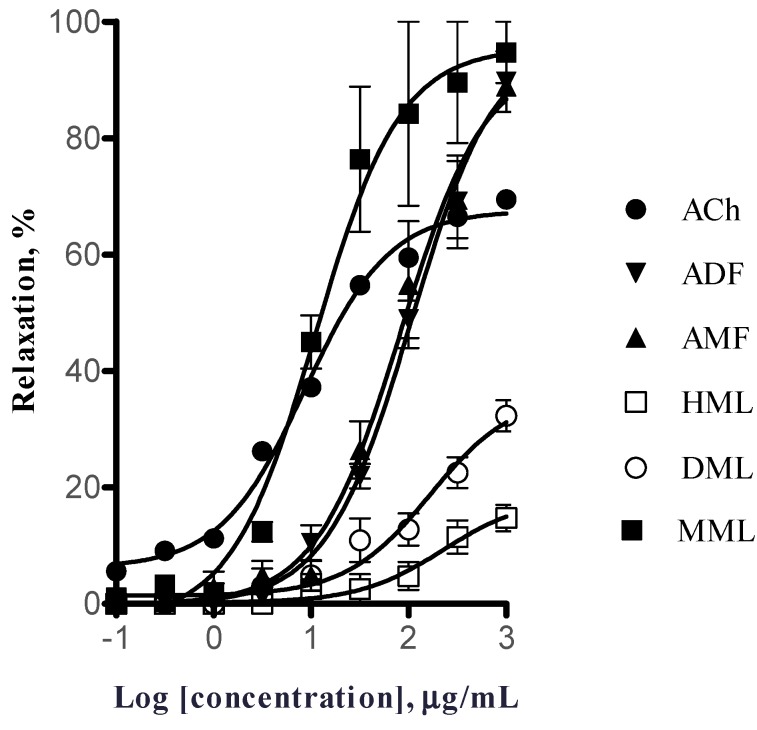
Concentration-response curves of the extracts obtained from the leaves and fruits of *C. gracilior* and ACh on isolated rat aorta rings precontracted with phenylephrine (PE) (1 µM). ACh = acetylcholine; ADF = aqueous decoction of the fruits; AMF = aqueous maceration of the fruits; HML = hexane maceration of the leaves; DML = dichloromethane maceration of the leaves; MML = Methanol maceration of the leaves.

All extracts elicited a concentration-dependent relaxation of aortic rings. Figure 1 shows the concentration-response curves for the five extracts and acetylcholine (Ach), which was used as a positive control. The values of EC_50_ and E_max_ are presented in Table 1.

The methanol extract obtained by maceration of *C. gracilior* leaves (MML) elicited the most potent vasorelaxant response (EC_50_ = 8.69 ± 4.34 µg/mL), which was similar to that of ACh (EC_50_ = 8.70 ± 0.80 µg/mL); however, this extract induced a higher E_max_ (94.60% ± 11.30%) than the positive control (E_max_ = 69.5% ± 5.70%). The aqueous extracts obtained from the fruits by decoction (ADF) and maceration (AMF) produced similar vasorelaxant effects and were approximately 10-fold less potent than that of MML extract. Finally, leaves extracts prepared by maceration with dichloromethane (DML) and hexane (HML) induced a very low maximum vasorelaxant response (less than 40%) and were even less potent than fruit extracts (Figure 1 and Table 1). These results show that methanol extract from *C. gracilior* leaves elicits a significant smooth muscle relaxation on rat aorta. Of all the extracts we evaluated, only MML showed a potency (EC_50_ = 8.69 ± 4.34 µg/mL) similar to that exhibited by the most popular drug WS1442 (7.4 ± 1.7 µg/mL) [5]. However, it was more potent than that of another commercial extract prepared with a mixture of *C. oxyacantha* and *C. monogyna* (batch No. 30365.01, containing more than 2% hyperoside) (EC_50_ = 27.5 µg/mL), that is also used in Europe as a herbal medicine to treat heart failure, angina pectoris and hypertension [18].

**Table 1:** Vasorelaxant effects induced by the extracts and some fractions of *C. gracilior* on the rat aortic rings precontracted with PE (1 µM).

*C. gracilior* Extracts or Fractions	% Relaxation *	Concentration at Maximal Effect (µg/mL)	EC_50_ (µg/mL) *
**Extracts**			
AMF	93.83 ± 13.12 ^a^	1000	83.14 ± 27.22 ^a^
ADF	96.89 ± 10.54 ^a^	1000	104.80 ± 23.80 ^ac^
MML	94.60 ± 11.30 ^a^	1000	8.69 ± 4.34 ^b^
DML	37.58 ± 10.72 ^be^	1000	206.00 ± 74.91 ^ae^
HML	18.52 ± 8.19 ^b^	1000	237.10 ± 51.74 ^ad^
**Fractions**			
F8	54.07 ± 2.51 ^de^	3160	316.30 ± 1.21 ^af^
F9	89.19 ± 12.76 ^ac^	3160	690.9 ± 1.75 ^cdf^
**ACh (control)**	69.50 ± 5.70 ^cd^	1000	8.70 ± 0.80 ^b^

* Values are mean ± S.E.M., *n* = 3 independent experiments performed in triplicates, and were determined by linear regression analysis using GraphPad Prism 5.01 Software; ^a–f^ Values are statistically significant at *p* ˂ 0.05.

**Figure 2:**
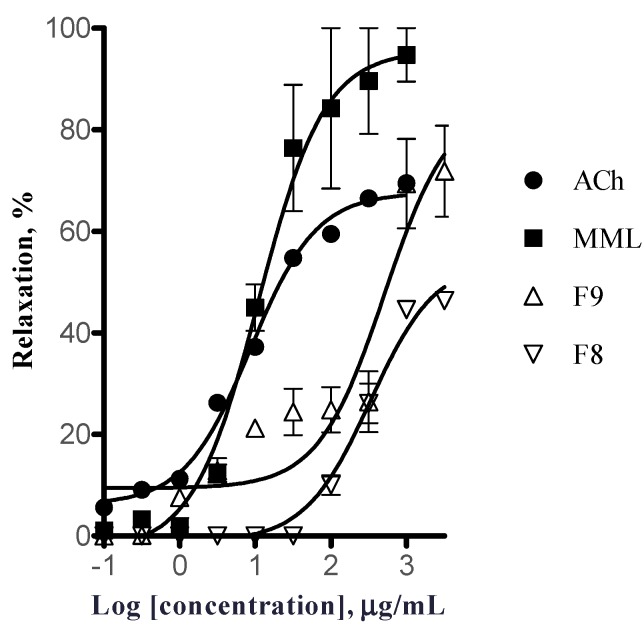
Vasorelaxant effects of fractions, MML extract and ACh on isolated rat aorta precontracted with PE (1 µM). ACh = acetylcholine; MML = methanol maceration of the leaves; F9 = fraction 9 (eluted with MeOH–acetone (20:80); F8 = fraction 8 eluted with CHCl_3_–MeOH (95:5).

According to the chromatographic profile and mass yields, the vasorelaxant activity of the fractions F8 and F9 were evaluated using the isolated rat aortic ring assay. Both fractions dilated the rings in a concentration-dependent manner (Figure 2). However, the vasorelaxant effect of these fractions decreased considerably in comparison with the effect elicited by MML (Table 1).

These results suggest that the higher vasorelaxant effect produced by MML extract is caused by different vasorelaxant compounds contained in the whole extract, which elicit their vasoactive effect in a synergistic manner. In the present study, ursolic acid was identified as the major compound in F8, which may account for the vasorelaxant effect of this fraction, since this triterpene induced an endothelium-dependent vasodilation (IC_50_ = 44.15 µM = 20.2 µg/mL) that seemed to be mediated by the release of nitric oxide from vascular endothelial cells [17]. The lower vasodilation elicited by F8 (15-fold less potent than ursolic acid) can be attributed to differences in methodological protocols, such as the vasoconstrictors used to contract the aorta. Additionally, it is possibly that F8 contains other compounds, which decrease the vasorelaxant effect produced by this triterpene. Also, corosolic acid was found to prevent oxidative stress, inflammation and hypertension in SHR/NDmcr-cp rats, a model of metabolic syndrome [19].

### 2.2. Identification and Quantification of Phenolic Compounds

**Figure 3:**
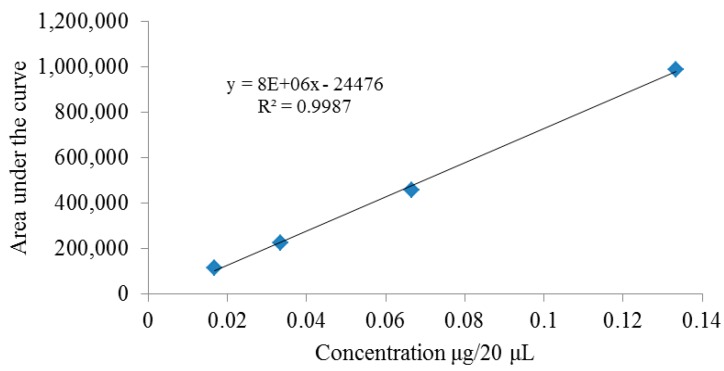
Densitogram obtained during quercetin quantification.

The most important vasorelaxant extracts (aqueous and methanol) of *C. gracilior* were subjected to a chemical study. As polar extracts are more likely to contain polar compounds like those used to standardize all commercial *Crataegus* preparations, ten common flavonoids and five phenolic acids were investigated in both non-hydrolyzed and hydrolyzed aqueous and methanol extracts using an HPLC chromatographic system. Their identification was achieved by comparison of their retention times (*Rt*) in each sample and those produced by authentic standards, as well as their respective UV spectra and co-chromatography. Chlorogenic acid (5-caffeoylquinic acid), rutin, (+)-catechin, kaempferol and quercetin (Figure 3) were identified and quantified using densitograms. As an example, Figure 3 shows the calibration curve obtained during quercetin quantification. The remaining densitograms can be found in the Supplementary Materials.

Chlorogenic acid was found in all the non-hydrolyzed extracts, although its low quantity in the macerate of the fruits fell beyond the calibration curve obtained during the experimental conditions. The bioside rutin was detected and quantified only in the methanol extract of the leaves (Table 2). Three of the phenolics were present as glycosides, as they were found only after acid hydrolysis of the methanol extract. (+)-Catechin gave the highest quantity, with 717.9 µg/g dry leaves. Acid hydrolysis of the fruits extracts revealed no additional compounds, indicating that none of the aglycones shown in the methanol extract was in these extracts. However, this fact does not rule out other phenolic compounds like procyanidins to be present.

**Table 2:** Contents and experimental *Rt* and λ_max_ of the phenolics identified in dry leaves and fresh fruits of *C. gracilior*.

		Phenolics Identified	*Rt* (min)	Experimental λ_max_ (nm)	Yields (µg/g Dry Leaves or Fresh Fruits)
Non hydrolyzed extracts	Methanol extract (MML)	Rutin	17.23	254.8353.7	300.8 ± 5.9
		Chlorogenic acid	13.99	325	35.1 ± 1.4
	Fruits decoction (ADF)	Chlorogenic acid	14.29	214.6323.8	3.3 ± 0.69
	Fruits maceration (AMF)	Chlorogenic acid	14.42	213.4322.6	N.Q.
Acid-hydrolyzed metanol extract		(+)-Catechin	14.61	278.5	717.9 ± 24.9
		Quercetin	22.810	254.8264.2	134.3 ± 2.1
		Kaempferol	21.168	265.4364.2	8.6 ± 0.2

Yields of phenolics are reported as mean ± SD of three replicates; N.Q. = not quantified.

Much attention has been paid in the recent years to flavonoids owing to their beneficial health effects, in particular for the prevention and treatment of cardiovascular diseases. Quercetin has been recognized to improve endothelium-dependent vasorelaxation in aorta and decreases systolic blood pressure and cardiac hypertrophy [20,21]. It has also demonstrated that it is able to regulate dyslipidemia [22]. These and other effects of quercetin support its therapeutic potential for hypertension and atherosclerosis treatments and prevention [23,24,25,26]. In the case of kaempferol, this flavone reduces and prevents arteriosclerosis by the inhibition of LDL oxidation and endothelial damage, as well as formation of platelets. It also increases endothelium-dependent vasorelaxation. Rutin, catechin and chlorogenic acid also exert roughly the same beneficial effects on cardiovascular diseases [15,17]. 

### 2.3. Purification and Identification of Ursolic and Corosolic Acids

**Figure 4:**
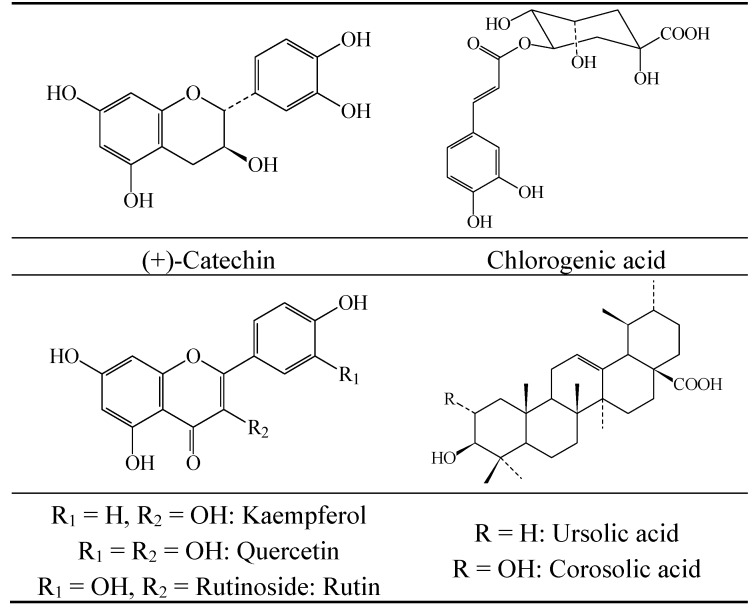
Structures of the compounds identified in the leaves and fruits of *C. gracilior*.

The most active extract (MML) was subjected to open column chromatography eluted with a stepwise gradient of hexane, dichloromethane, chloroform, acetone and methanol. Eleven pooled fractions were obtained. TLC of fraction 8, eluted with CHCl_3_–MeOH (95:5), revealed a less complex chemical profile. Preparative TLC of this fraction afforded ursolic acid (17.0 mg) and corosolic acid (8.5 mg), two of the triterpenes already identified in some *Crataegus* species (Figure 4). Ursolic acid was first identified by analytical TLC where an authentic standard was used, but the final identification of these two acids was achieved through the study of their NMR data recorded in pyridine. These data matched entirely those reported in the literature [27,28]. Two other triterpenes (CG-AB1: 12.0 mg and CG-AB4: 6.0 mg) were isolated, although it was not possible to complete their structure elucidation because many attempts to achieve their optimal purification failed. However signals observed in their ^1^H and ^13^C-NMR spectra corresponded to the pentacyclic skeleton of triterpenes like those of ursolic and corosolic acids. The corresponding spectra can be found in the Supplementary Materials.

## 3. Experimental Section

### 3.1. General Information

HPLC separations were conducted using a Waters apparatus (Millipore Corp., Waters Chromatography Division, Milford, MA, USA), composed of a 600E multi solvent delivery system and a 2998 PDA detector. Control of this equipment, data acquisition, processing, and management of the chromatographic information were performed by the Empower v2 software (Waters). Nuclear magnetic resonance (NMR) spectra were taken on a Varian VNMRS 400 spectrometer (Varian NMR Inc., Palo Alto, CA, USA) with tetramethylsilane (TMS) as internal standard, and chemical shifts are expressed in δ (symbol) values (ppm). Electron Impact Mass Spectrometry (EIMS and negative FAB) measurements were carried out on a Thermo Electron DFS (Double Focus Sector) spectrometer (Thermo Fisher Scientific Inc., Waltham, MA, USA). The isometric tension was measured on a Grass FT03 force displacement transducer attached to a Grass 7D polygraph (Grass Products, Warwick, RI, USA). Merck Si-gel plates were used for the purification of triterpenes. 

### 3.2. Chemicals

Phenylephrine hydrochloride (PE) and acetylcholine (ACh), as well as phenolic standards were obtained from Sigma Chemical Co. (St. Louis, MO, USA). HPLC and analytical solvents were purchased from Baker-Mallincrodt (JT Baker, Mallinckrodt Baker Inc, Phillipsburg, NJ, USA).

### 3.3. Plant Materials

The leaves and fruits from *C. gracilior* were collected in Pinal de Amoles, Qro., Mexico, during November 2012. A voucher specimen was authenticated and deposited in the Ethno-botanical Collection of the Herbarium of Querétaro “Dr Jerzy Rzedowski”, located at the Faculty of Natural Sciences, University of Querétaro, Mexico (voucher specimen: A. Cabrera 5667). The leaves were subjected to a drying process using an oven set at 40 °C, while the fruits were kept at −4 °C.

### 3.4. Extraction and Fractionation

Ground dried leaves (1.6 kg) were macerated successively with hexane, dichloromethane and methanol. The following quantities were obtained: ADF: 35.31 g (6.73%); AMF: 38.91 g (7.25%); HML: 6.10 g (0.37%); DML: 22.00 g (1.65%); MML: 201.3 g (13.77%). Each extract was evaporated until dry. The methanol extract was fractionated in an open CC (silica gel Kiesegel 60 Merck, 70–230 mesh ASTM, particle size 0.063–0.200 mm), which was eluted with hexane, dichloromethane, acetone and methanol in an increasing stepwise. All fractions were monitored by TLC and pooled together into 11 fractions on the basis of their similarity. The pooled fraction 97–119 (F8) (12.16 g) eluted with chloroform–methanol (95:5, v:v) was subjected to preparative TLC on silica gel (Merck, 60F_254_, 20 × 20 cm, thickness 250 µm) and the plates were eluted twice with chloroform–methanol (95:5, v:v). On the other hand, F9 (eluted with MeOH–acetone 20:80), was obtained with a yield of 23.06 g. The defrosted fruits (1.285 kg) were first separated from their seeds and the pulp obtained (1.061 kg) underwent maceration and decoction with water. The aqueous extracts were freeze-dried and stored until chemical and *ex vivo* pharmacological studies. 

### 3.5. Triterpene Characterization

*Ursolic acid*: ^1^H-NMR (400 MHz, pyridine-d_5_) δ: 5.81 (1H, t; *J* = 3.6 Hz, H-12), 3.77 (1H, dd; *J* = 6.0 Hz; 10.0 Hz, H-3), 2.95 (1H, d; *J* = 11.2 Hz, H-18), 1.56 (3H, s, H-27), 1.54 (3H, s, H-23), 1.37 (3H, s, H-24), 1.34 (3H, s, H-26), 1.32 (3H, d; *J* = 6.4 Hz, H-29), 1.26 (3H, d; *J* = 6.0 Hz, H-30), 1.20 (3H, s, H-25).^13^C-NMR (100 MHz, pyridine-d_5_) δ: 180.0 (C-28), 139.3 (C-13), 125.7 (C-12), 78.2 (C-3), 55.9 (C-5), 53.7 (C-18), 48.1 (C-9), 48.1 (C-17), 42.6 (C-14), 40.1 (C-8), 39.6 (C-4), 39.5 (C-19), 39.5 (C-20), 39.2 (C-1), 37.5 (C-10), 37.4 (C-22), 33.7 (C-7), 31.1 (C-21), 28.9 (C-23), 28.8 (C-15), 28.2 (C-2), 25.0 (C-16), 24.0 (C-27), 23.7 (C-11), 21.5 (C-30), 18.9 (C-6), 17.6 (C-26), 17.5 (C-29), 16.7 (C-24), 15.8 (C-25). These experimental data were compared with literature [27]. EI-MS (*m*/*z*): 456 (M+), 438, 411, 410, 392, 390, 377, 349, 249, 248 (100%), 219, 207, 203, 189, 133, 119, 105, 95, 93, 55, 43. 

*Corosolic acid*: ^1^H-NMR (400 MHz, pyridine-d_5_) δ: 5.77 (1H, t; *J* = 3.6 Hz, H-12), 4.09 (td; *J* *=* 10.2 Hz; 4.2 Hz, H-2), 3.71 (1H, d; *J* = 9.2 Hz, H-3), 2.93 (1H, d; *J* = 11.2 Hz, H-18), 1.27 (3H, s, H-23), 1.20 (3H, s, H-27), 1.07 (3H, s), 1.04 (3H, s, H-24), 0.97 (3H, s, H-25), 0.97 (3H, d, *J* = 6.4 Hz, H-29), 0.94 (3H, d, *J* = *6*.0 Hz, H-30). ^13^C-NMR (100 MHz, pyridine-d_5_) δ: 180.0 (C-28), 139.8 (C-13), 126.0 (C-12), 84.3 (C-3), 69.1 (C-2), 56.4 (C-5), 48.5 (C-1), 40.3 (C-4), 38.9 (C-10), 34.0 (C-7), 29.1 (C-15), 19.3 (C-6), 17.3 (C-25). These experimental data were compared with literature data [28]. Negative FAB-MS: 471 (M - H) (100%), 469, 453, 439, 437, 423, 355, 325, 311, 265, 243, 183, 91, 59.

### 3.6. Acid and Base Hydrolysis of Methanol and Aqueous Extracts

One hundred mg of the methanol and aqueous extracts obtained respectively from leaves and fruits were separately placed in a flask, and 10 mL of MeOH or water (HPLC grade) were added. Five mL of 2 N HCl or 2 N NaOH were then added, and the mixtures were subjected to reflux for 2 h. Twenty µL of these reaction mixtures were analyzed by the HPLC-PDAD system after filtration on acrodiscs (45 µm pore size and 25 mm diameter, Agilent Technologies, Santa Clara, CA, USA). 

### 3.7. HPLC Determination of Phenolics

A standardized method developed in our lab was used for phenolics identification and quantification. The following analytical conditions were used: column: ZORBAX Eclipse XDB-C18 (3.5 μm, 4.6 × 150 mm i.d.) provided with a pre-column (Agilent Technologies); mobile phase: linear gradient of 0.0125 N aqueous-acetic acid (eluent A) and CH_3_CN (eluent B), starting from 95% A and reaching 50% in 20 min and then returning to 95% from 20 min to 25 min, composition which was then maintained until 35 min; the flow rate was 0.7 mL/min and the injection volume 20 μL; peaks were detected at 280 nm. 

### 3.8. Quantification of the Identified Phenolics

For the quantification of the identified phenolics, densitograms were obtained using the following concentrations (μg/20 μL): (+)-catechin: 0.320, 0.427, 0.533 y 0.640; quercetin: 0.017, 0.033, 0.067 y 0.133; kaempferol: 0.009, 0.007, 0.004 y 0.004; rutin: 0.040, 0.053, 0.080 y 0.160; chlorogenic acid: 0.029, 0.015, 0.010 y 0.007. Four points were obtained, each one the mean of three replicates. The areas under the curves (A) obtained for each standard were plotted *versus* the respective concentrations used (x) and a linear correlation established as A = ax + b. Then, the amounts of each phenolic identified was calculated by intrapolation in the densitogram and then scaled up to extracts. 

### 3.9. Determination of the Vasorelaxant Effect

For the measurement of vasorelaxant effects, the two aqueous extracts from *C. gracilior* fruits (AMF and ADF) were dissolved in distilled water. The methanol extract obtained from the leaves (MML) was dissolved in DMSO, while the hexane (HML) and dichloromethane (DML) extracts were dissolved firstly in Tween 80 and then diluted in distilled water. Maximal concentration of Tween and DMSO in samples was less than 0.2% (*v*/*v*), which had no effect on tension development of isolated aorta. All experiments were performed in accordance with The Mexican Official Standard NOM-062-ZOO-1999 for the production, care, and use of laboratory animals [29]. The vasorelaxant effect was measured on the isolated rat aortic ring assay as described by Ibarra-Alvarado *et al.* [30]. Briefly, male Wistar rats weighing 250 to 300 g were anesthetized with chloroform and sacrificed by decapitation and the descending thoracic aorta removed, placed in ice-cold oxygenated Krebs-Henseleit solution of the following composition: 126.8 mM NaCl, 5.9 mM KCl, 2.5 mM CaCl_2_, 1.2 mM MgSO_4_, 1.2 mM KH_2_PO_4_, 30 mM NaHCO_3_, and 5 mM d-glucose (pH 7.4) and flushed to prevent clot formation. Adipose and connective tissues were removed and the aorta sliced at 4–5 mm intervals into rings. The aortic rings were mounted between stainless steel hooks and suspended in water-jacketed, 7 mL organ baths containing oxygenated (95% O_2_ and 5% CO_2_) Krebs-Henseleit solution at 37 °C. The tissues were allowed to equilibrate for 60 min under a resting tension of 1.5 g. During this period, the bathing medium was exchanged every 15 min. After final adjustment of the passive resting tension to 1.5 g, aortic segments were contracted with 100 mM KCl. Once a stable contractile tone was reached, the bathing medium was exchanged to restore a resting tension of 1.5 g. After that, tissues were contracted with 1 µM L-phenylephrine (PE) with force of contraction recorded and defined as 100%. The endothelial integrity of the preparations was determined by adding acetylcholine (1 µM) to the superfusate. Only arteries with a vasodilator response of >70% inhibition of preconstriction were considered endothelium-intact. The crude extracts or acetylcholine (ACh) were added to the bath at final concentrations of 0.1–1000 µg/mL, while the fractions F8 and F9 at final concentrations ranging from 0.1 μg/mL to 3160 μg/mL. Responses were expressed as percent initial contraction achieved with PE. 

### 3.10. Statistical Analysis

Results of the experiments were expressed as the mean ± S.E.M. from *n* = 3 independent experiments performed in triplicates. Concentration-response curves (CRCs) for the tested substances were plotted and fitted to the sigmoidal concentration-response equation using the data analysis and graphics program Prism 5.0 (GraphPad Software, San Diego, CA, USA). The values of the mean effective concentrations (EC_50_) and maximum vasorelaxant effects (E_max_) were obtained from the concentration-response curves. The data were analyzed by a one-way ANOVA and the Tukey test. Differences between the means were considered to be significant when *p* ˂ 0.05.

## 4. Conclusions

The results obtained in the present study demonstrate the vasorelaxant effect of the MeOH and aqueous extracts of the medicinal plant *Crataegus gracilior* and led to the identification of some of its bioactive compounds, which have already demonstrated their beneficial effects on cardiovascular diseases. In that way, they support one of its ethnomedical uses and open the perspective of developing another standardized extract as an aid in the treatment of at least hypertension. 

## Supplementary Materials

Supplementary materials can be accessed at: http://www.mdpi.com/1420-3049/19/12/20962/s1.
