# Supplementary material for: Aortic Relaxant Activity of Crataegus gracilior Phipps and Identification of Some of Its Chemical Constituents
Source: Molecules. 2014 Dec 15;19(12):20962–74. doi: 10.3390/molecules191220962 (PMC6272000; doi:10.3390/molecules191220962)

Supplementary Materials

Figure S1. Experimental densitogram obtained for kaempferol quantification.

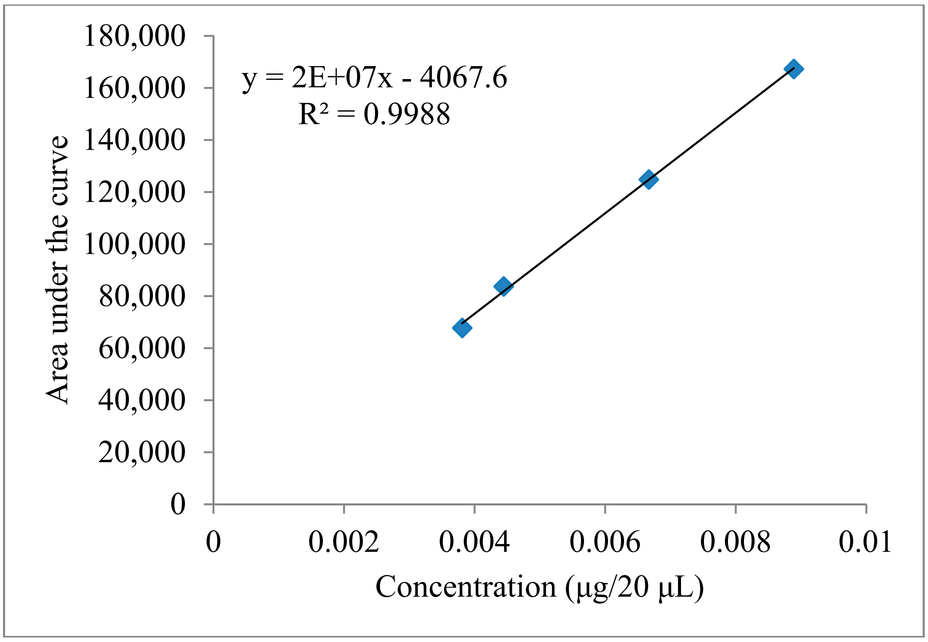

Figure S2. Experimental densitogram obtained for (+)-catechin quantification.

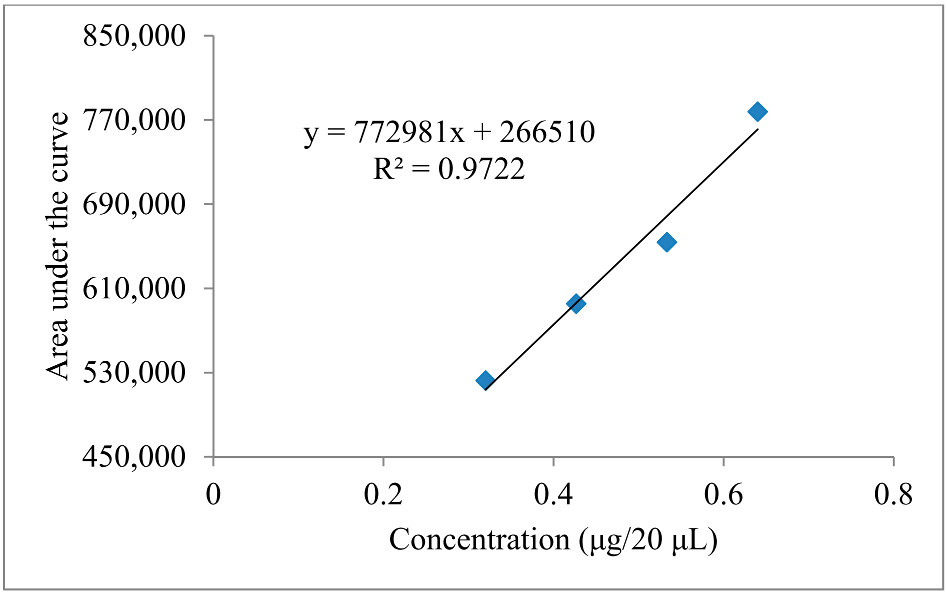

Figure S3. Experimental densitogram obtained for rutin quantification.

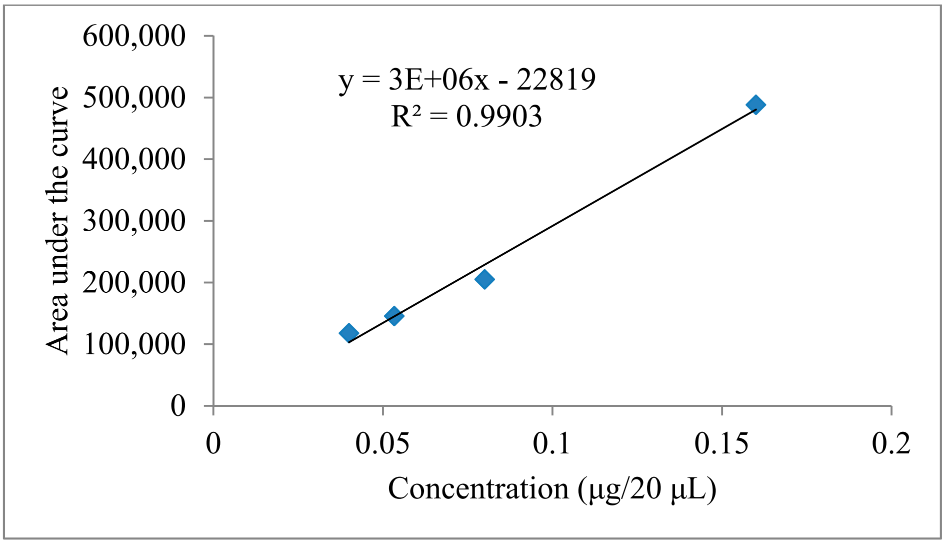

Figure S4. Experimental densitogram obtained for chlorogenic acid quantification.

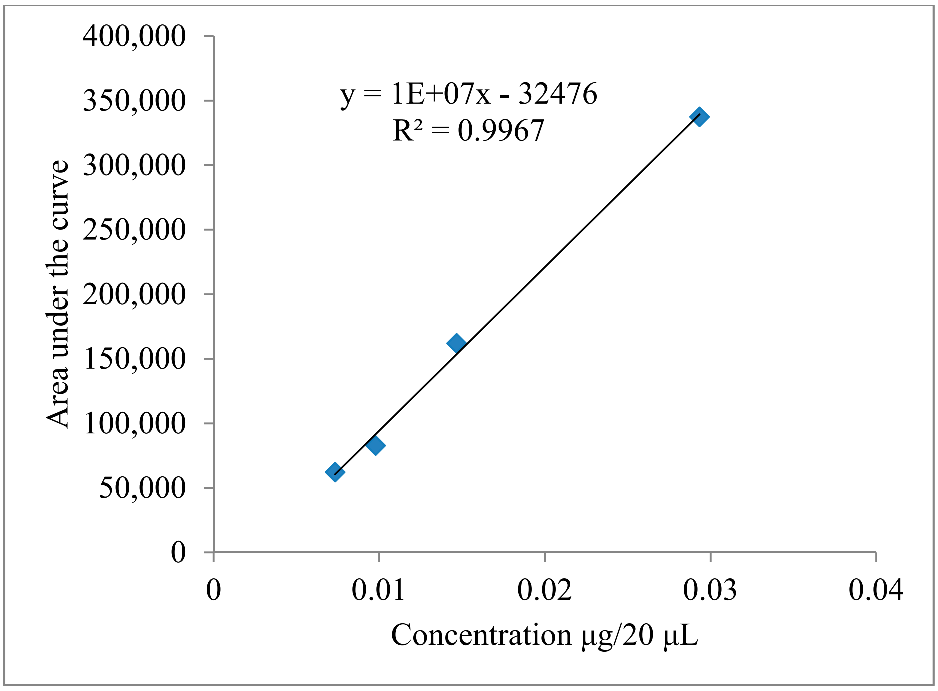

Figure S5. ¹H-NMR spectrum of ursolic acid (400 MHz, pyridine-d_5).

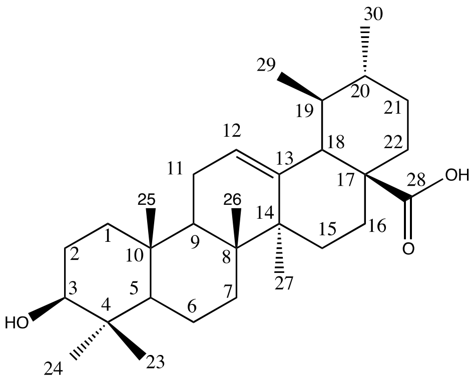

Figure S5. *Cont.*

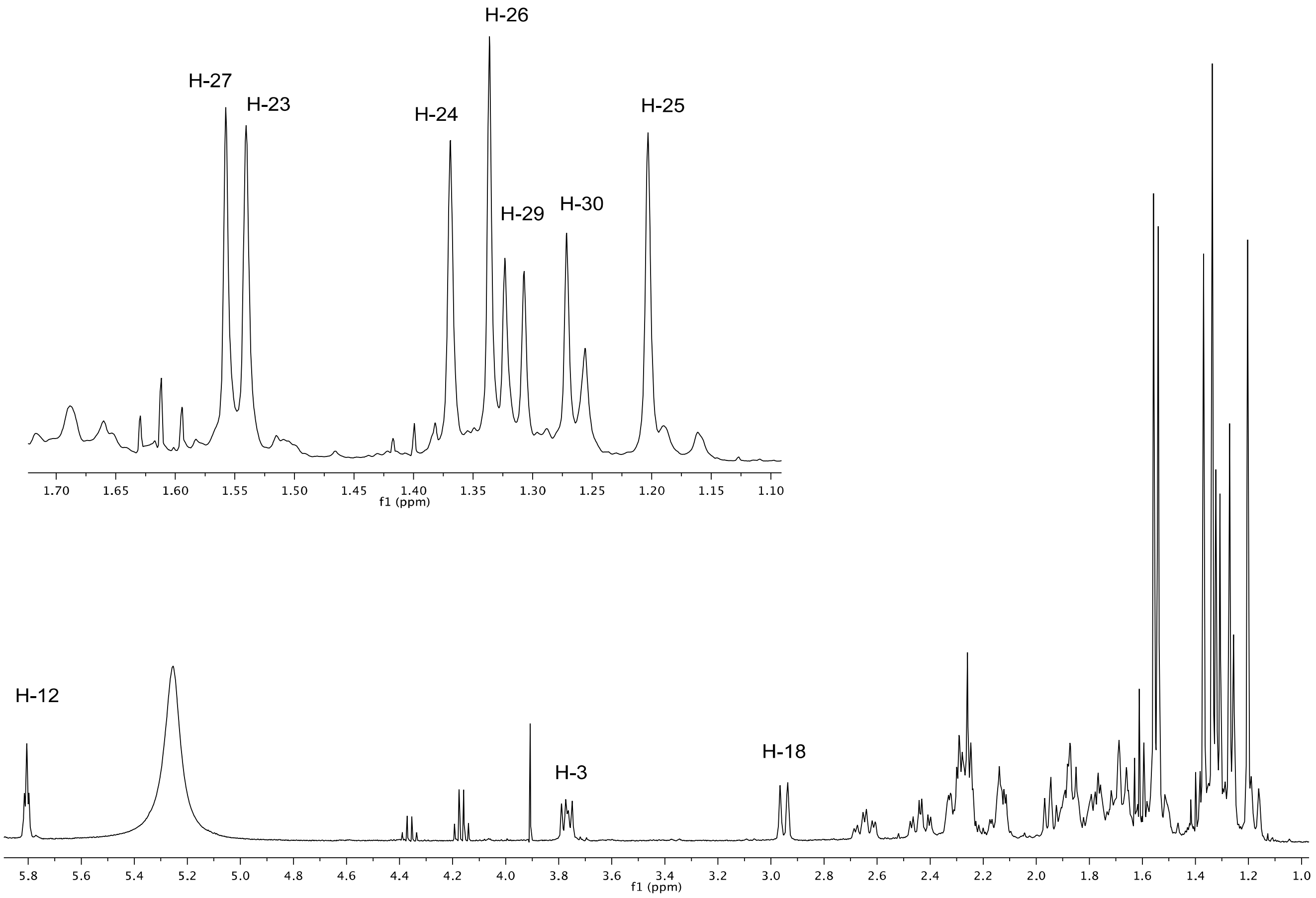

Figure S6. ^{13}C-NMR spectrum of ursolic acid (100 MHz, pyridine-d_5).

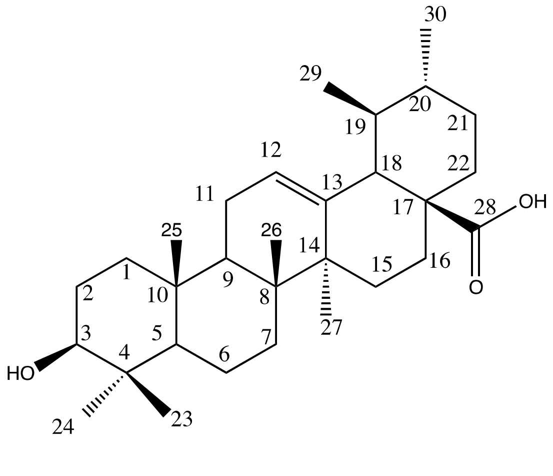

Figure S6. *Cont.*

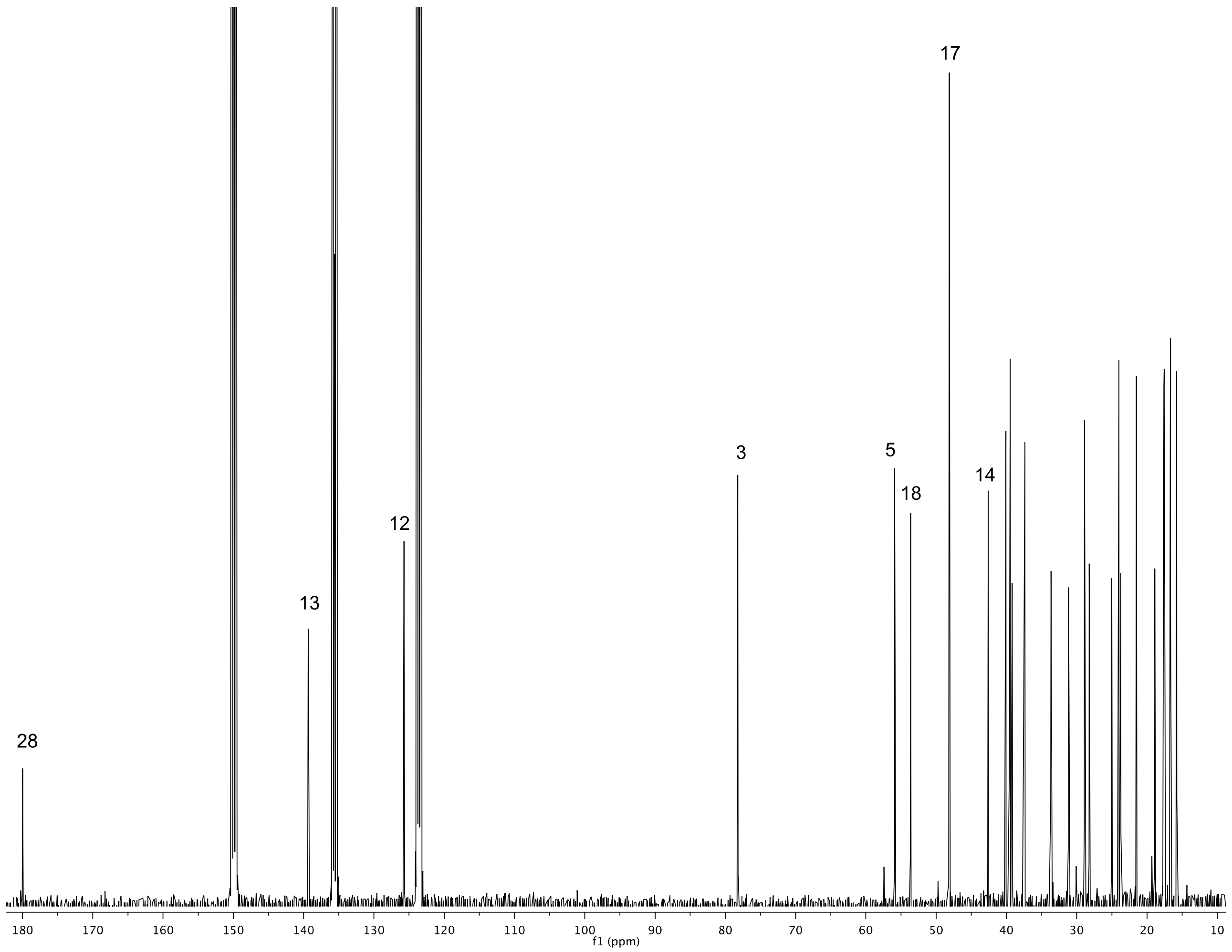

Figure S7. EI-MS of ursolic acid.

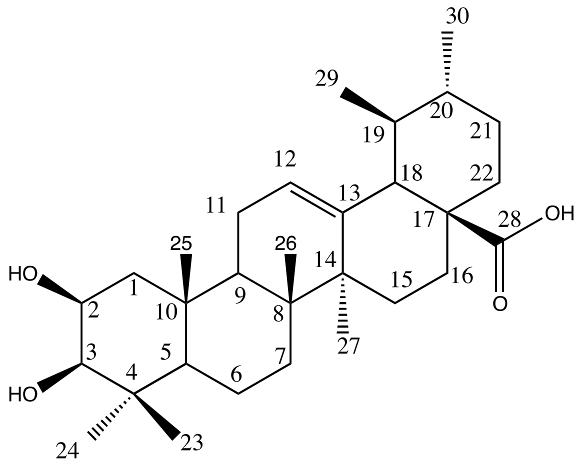

Figure S7. *Cont.*

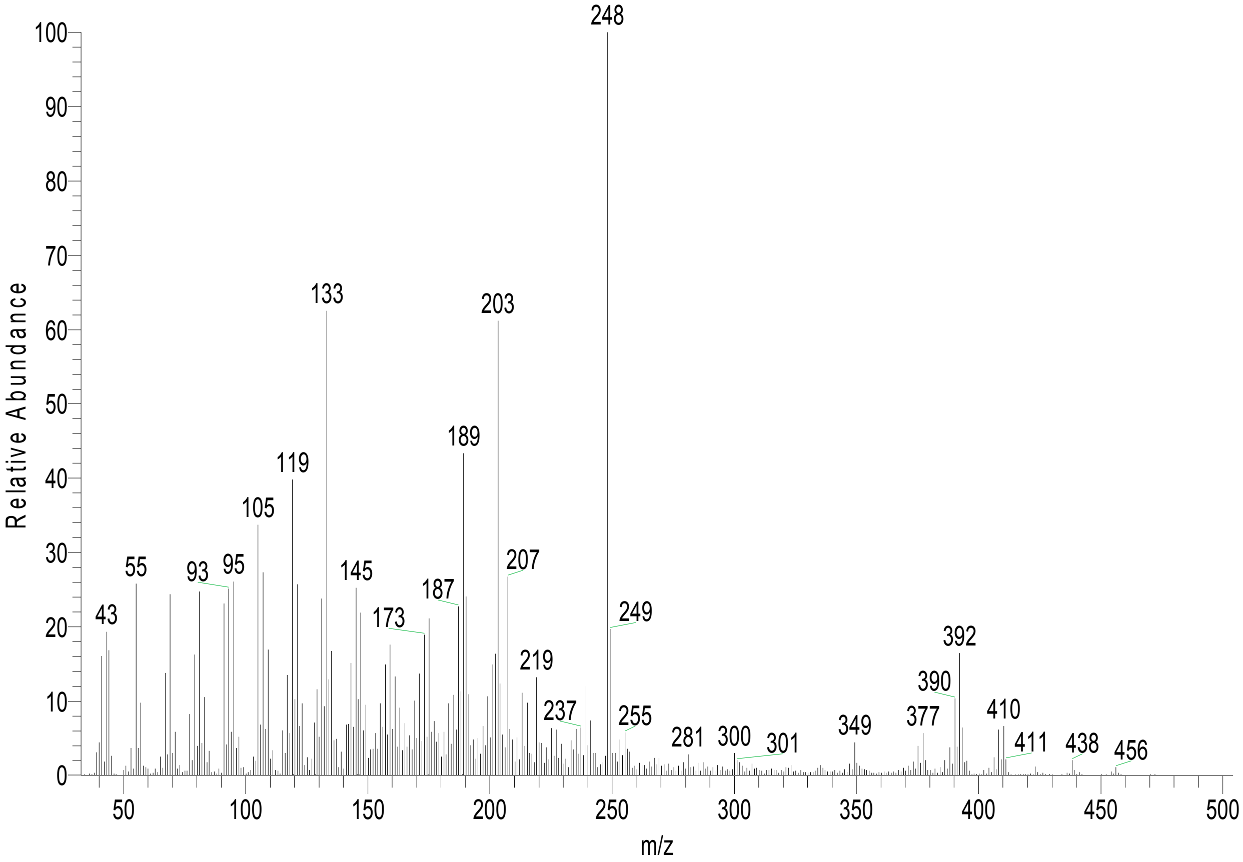

Figure S8. ^{1}H-NMR spectrum of corosolic acid (400 MHz, pyridine-d_5).

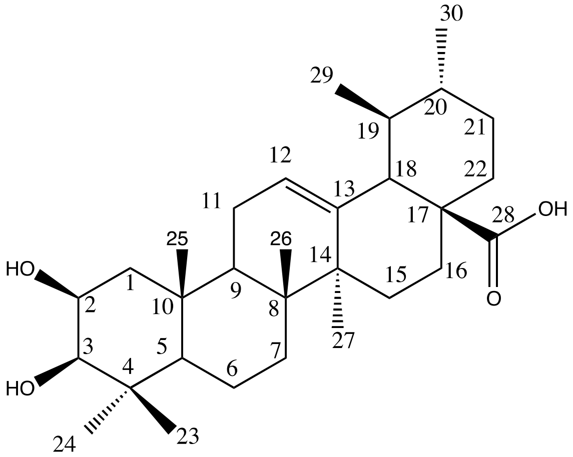

Figure S8. *Cont.*

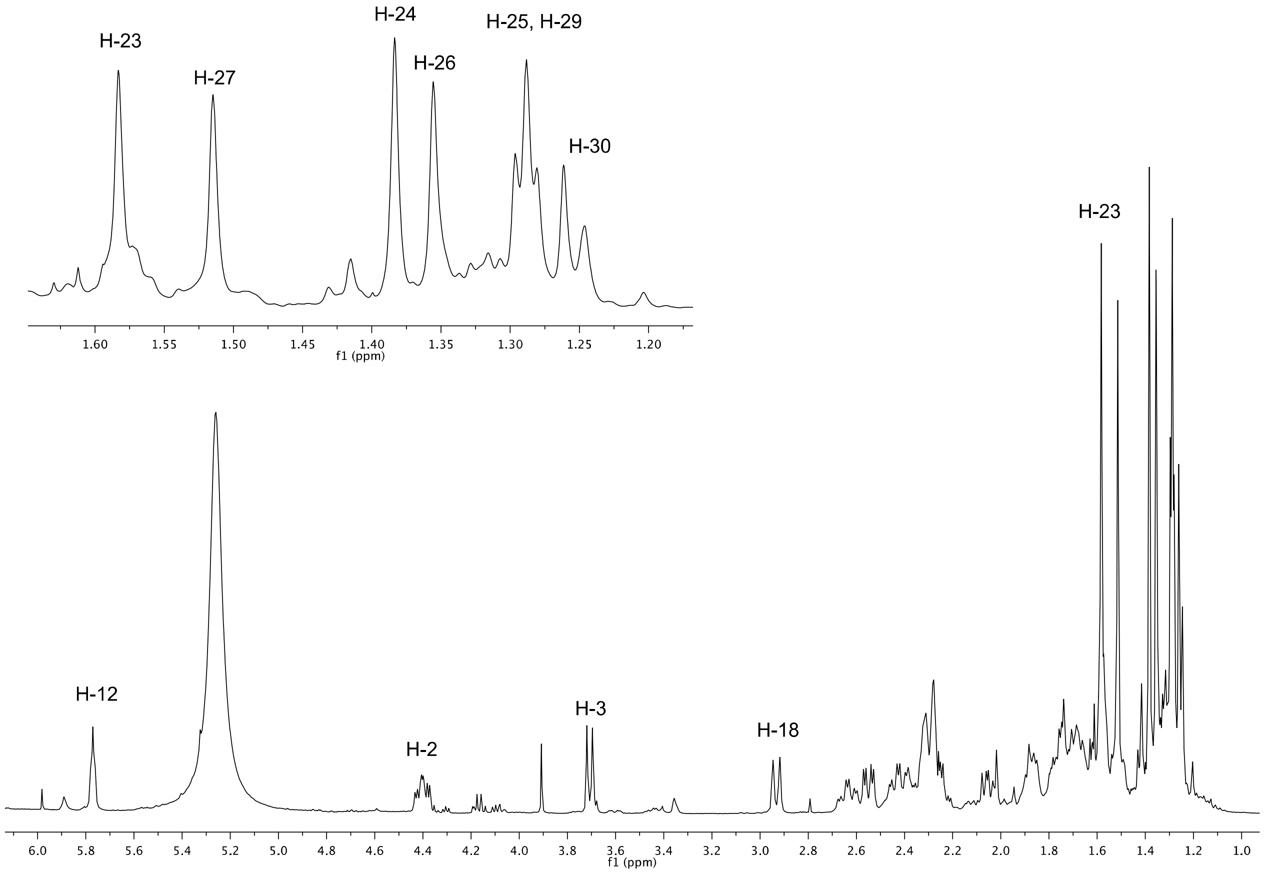

Figure S9. ^{13}C-NMR spectrum of corosolic acid (100 MHz, pyridine-d_5).

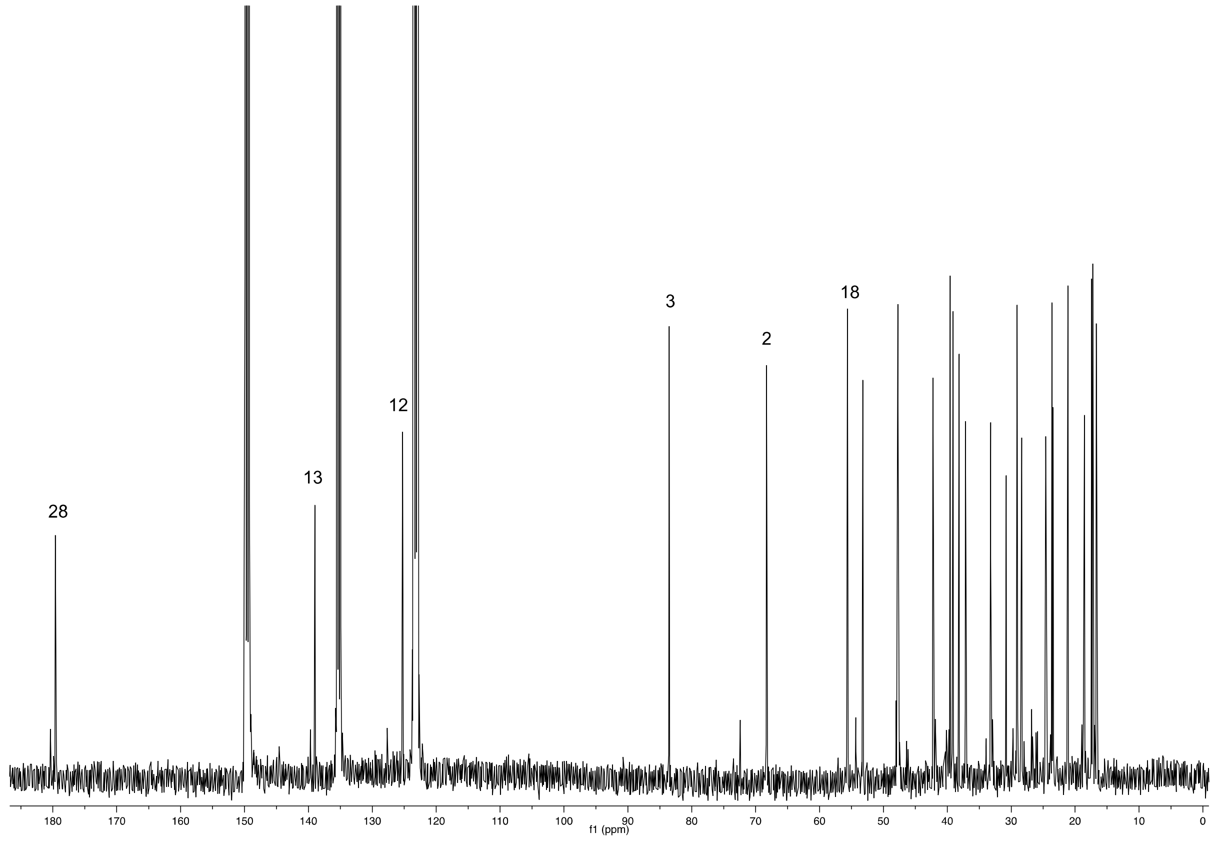

Figure S10. Negative FAB-MS of corosolic acid.

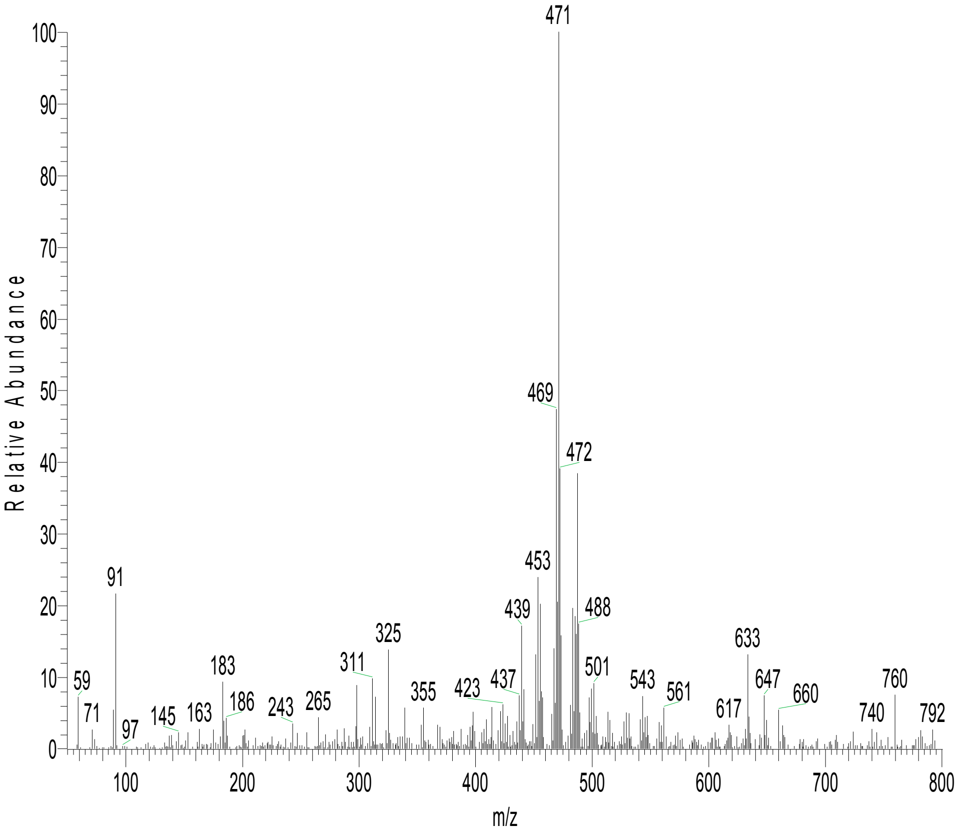

Figure S11. ^{1}H-NMR spectrum of compound CG-AB1 (400 MHz, DMSO-d_6).

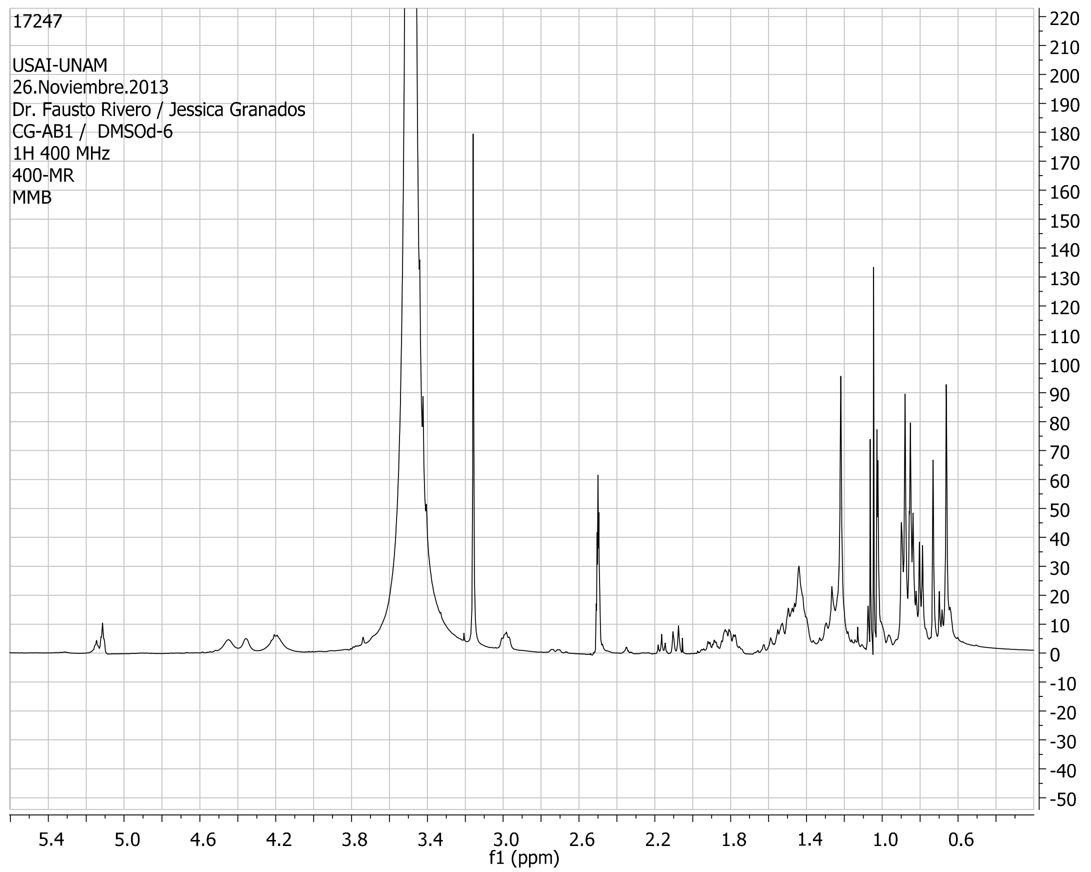

Figure S12. ^{13}C-NMR spectrum of non-identified compound CG-AB1 (100 MHz, DMSO-d_6).

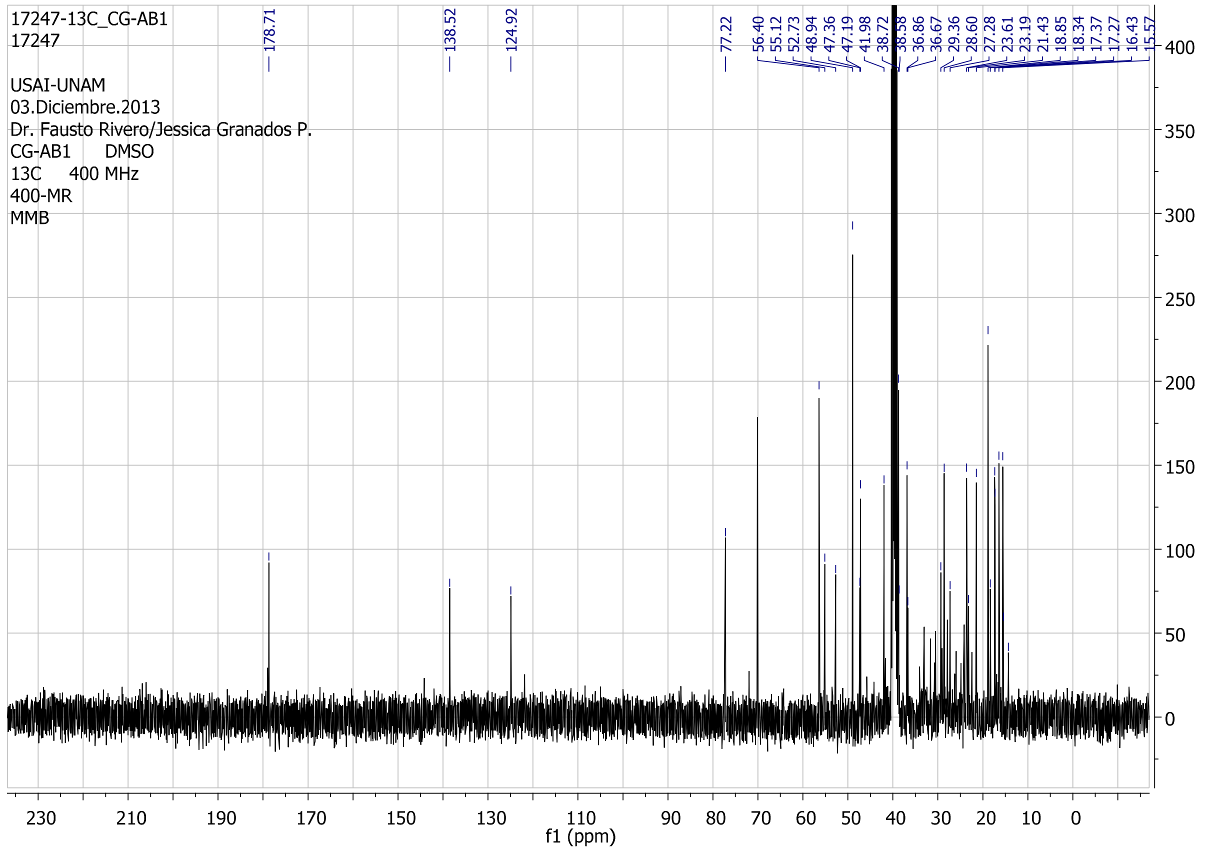

Figure S13. ^{1}H-NMR spectrum of non-identified compound CG-AB4 (100 MHz, DMSO-d_6).

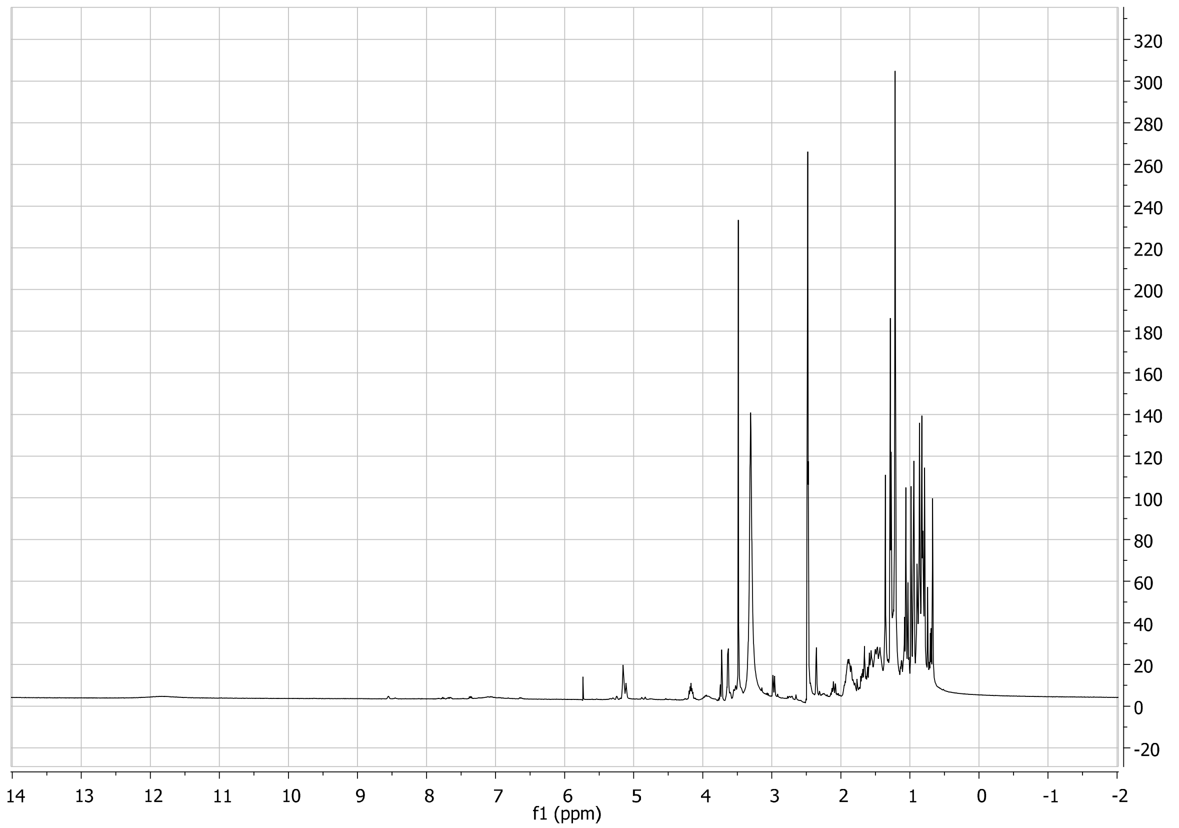

Figure S14. ^{13}C-NMR spectrum of non-identified compound CG-AB4 (100 MHz, DMSO-d_6).

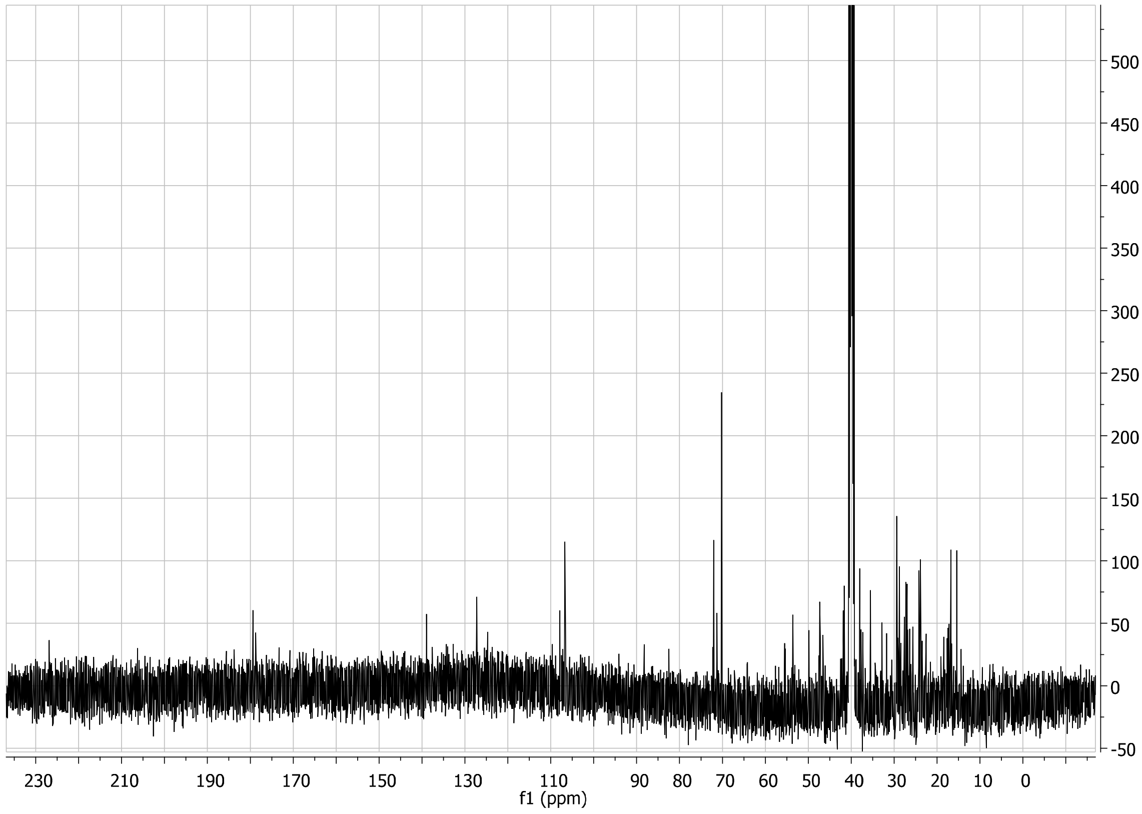

Supplement: Supplementary file 1 [file molecules-19-20962-s001.pdf]
